# Successive Oral Immunizations Against *Piscirickettsia Salmonis* and Infectious Salmon Anemia Virus are Required to Maintain a Long-Term Protection in Farmed Salmonids

**DOI:** 10.3389/fimmu.2015.00244

**Published:** 2015-05-27

**Authors:** Iván Tobar, Sergio Arancibia, Constanza Torres, Verónica Vera, Paola Soto, Claudia Carrasco, Marcelo Alvarado, Eduardo Neira, Sandra Arcos, Jaime A. Tobar

**Affiliations:** ^1^Department of Research and Development, Virbac-Centrovet, Santiago, Chile

**Keywords:** salmonids, oral vaccination, SRS, ISAv, long-term protection, field conditions

## Abstract

Currently, there is a growing demand to determine the protective status of vaccinated fish in order to prevent diseases outbreaks. A set of different parameters that include the infectious and immunological status of vaccinated salmonids from 622 Chilean farms were analyzed during 2011–2014. The aim of this study was to optimize the vaccination program of these centers through the determination of the protective state of vaccinated fish using oral immunizations. This state was determined from the association of the concentration of the immunoglobulin M (IgM) in the serum and the mortality rate of vaccinated fish. Salmonids were vaccinated with different commercial mono- or polyvalent vaccines against salmonid rickettsial septicemia (SRS) and infectious salmon anemia (ISA), first by the intraperitoneal injection of oil-adjuvanted antigens and then by the stimulation of mucosal immunity using oral vaccines as a booster vaccination. The results showed that high levels of specific IgM antibodies were observed after injectable vaccination, reaching a maximum concentration at 600–800 degree-days. Similar levels of antibodies were observed when oral immunizations were administrated. The high concentration of antibodies [above 2750 ng/mL for ISA virus (ISAv) and 3500 ng/mL for SRS] was maintained for a period of 800 degree-days after each vaccination procedure. In this regard, oral immunizations maintained a long-term high concentration of anti-SRS and anti-ISAv specific IgM antibodies. When the concentration of antibodies decreased below 2000 pg/mL, a window of susceptibility to SRS infection was observed in the farm, suggesting a close association between antibody levels and fish protective status. These results demonstrated that, in the field, several oral immunizations are essential to uphold a high level of specific anti-pathogens antibodies and, therefore, the protective status during the whole productive cycle.

## Introduction

The Salmon industry has significantly increased worldwide over the last two decades. In Chile, the industry has rapidly grown becoming one of the most important factors in the country’s development. Different diseases may impact on the immune balance of salmon that, eventually, will end up affecting its production. Methods such as vaccination have improved salmon production and have helped to prevent the excessive use of antibiotics as a means to prevent diseases outbreaks. *Piscirickettsia salmonis* and ISA virus (ISAv) provoke salmonid rickettsial septicemia (SRS) and infectious salmon anemia (ISA), respectively, and have caused severe outbreaks during the last 6 years in Chile, resulting in significant loss of salmon production. Vaccination against these pathogens in farmed salmonids is mostly administered using an inactivated bacteria or virus emulsified in an oil-adjuvanted preparation given via an intraperitoneal (i.p) injection. Recently, we have demonstrated that mucosal stimulation by oral vaccination against SRS is less stressful for fish, delivers strong protection and is very safety (1). This vaccination method is advantageous for both small-sized salmon and large-scale procedures, making it ideal for successive long-term immunizations during the sea-water growth stage. Although, different commercial vaccines against *P. salmonis* and ISAv present high degrees of protection under experimental conditions, the efficacy of vaccination in the field will depend on several factors such as the vaccination procedures used, the immunobiology of the fish, time of vaccination, and environmental factors (2, 3). The study of the fish’s immune response is an area of particular interest to the aquaculture industry, especially the duration of immunity after vaccination. One of the main problems to do this is the absence of information to indicate changes in the protection status of salmonids during the course of their production cycle.

The immune system of teleost fish has some similarities to that of mammals’, where immunoglobulins are one of the most important components of the immune response. In salmonids, most of the current vaccines protect through neutralizing antibodies, either to prevent the infection from spreading or to interfere the action of microbial products. Tetrameric immunoglobulin M (IgM) is the prevailing class in serum, which increases significantly upon infection or vaccination. The immunological protection after vaccination is often a relative aspect because it depends on several factors such as host response, infection threshold, and the complexity of the immune response (4). However, in most cases, a high concentration of specific IgM antibodies can be associated with a protective state in vaccinated fish. Indeed, several studies have been carried out to relate specific serum IgM levels and the protective capacity of vaccines against different pathogens (5–10). However, these reports only studied the efficacy of vaccination under experimental conditions, and none of them focused on the field studies, where several factors may affect fish immune response and protection elicited by the vaccine.

In the present work, we studied the protective state of vaccinated salmonids from several aquaculture farms in Chile, by examining the association between antibody levels after vaccination and mortality rate due to SRS. We analyzed, at various degree-days, specific serum IgM levels against *P. salmonis* and ISAv, in order to establish an appropriate vaccination program that indicates the minimum IgM concentration necessary to promote immunological protection and to avoid infectious outbreaks, especially against SRS and ISAv. In addition, we evaluated the use of oral immunization as a measure to maintain a high-IgM level along the entire productive cycle. We developed and used a quantitative IgM enzyme-linked immunosorbent assay (ELISA) to assess the levels of specific antibodies in the vaccinated fish.

## Materials and Methods

### Animal management and serum sampling

Salmonid fish were maintained by each farm and subjected to environmental factors specific to each geographical region of Chile (X, XI, and XIV–IX). All sampling procedures were authorized by the farming companies and performed according National Fisheries Services guidelines and supervised by veterinarians. Animal procedures were approved by Institutional CICUAL (Translation: Comité Institucional de Cuidado y Uso de Animales de Experimentación, Institutional Comiteé for care and use of Animals for experimentation), which follows international ethics guidelines and is composed of institutional experts and external advisers. Blood samples were collected by each farm by caudal venous puncture and then immediately transferred to a tube and left for 24 h at 4°C for serum extraction. The average number of sera obtained from each aquaculture center at a specific time point after vaccination was 10. Samples were transported according to proper biosafety procedures including appropriate containments, cold chain monitoring and laboratory reception, classification, and storage. Over 4 years, 32,399 serum samples were collected from 622 Chilean farms at different periods after vaccination. Forty-five percent of the sera were obtained from *Salmo salar*, 34% from *Oncorhynchus mykiss*, and 21% from *Oncorhynchus kisutch*. Most of sera were collected from sea-water farms (74%) although some were collected from freshwater (20%) and estuary (6%) farms. The average temperature of water of the farms located in the X, XI, and XIV–IX regions of Chile was 11.73 ± 0.13, 11.09 ± 0.14, and 15.54 ± 0.75°C, respectively. The field study did not involve endangered or protected species.

### Vaccination

The number of immunizations and type of vaccines utilized in the study varied between the farms; however, at least one injectable vaccine against SRS or ISAv was applied in all the farms as primary vaccination. Some of these farms also performed one or two oral vaccinations as booster immunization in order to maintain immunity against the disease. On the freshwater stage of growth, fish was first vaccinated by intraperitoneal (i.p) injection with an oil-adjuvanted commercial vaccine containing either *P. salmonis* or ISAv antigen. Fifty-five percent of the farms utilized mono- or polyvalent injectable vaccines provided by Virbac-Centrovet and the rest were from other pharmaceuticals companies. Virbac-Centrovet injectable vaccines were divided into monovalent (SRS – 9% of the farms applied this type of vaccine), mixed [SRS + infectious pancreatic necrosis (IPN) – 28%], triple (SRS + IPN + *Vibrio ordalii* – 9%), tetravalent (SRS + IPN + *V. ordalii* + ISAv – 17%), or pentavalent (SRS + IPN + *V. ordalii* + ISAv + *Aeromonas salmonicida* – 37%). The injectable vaccines of other pharmaceutical companies were categorized into mixed (29% of the farms applied this type of vaccine), triple (5%), tetravalent (24%), and pentavalent (39%). In the saltwater growth stage, fish was immunized once or two times by oral route during 10 consecutive days, as previously reported (1). The oral formulations used by the farms were mainly monovalent (SRS – 78% and ISAv – 14%) or mixed (SRS + ISAv – 8%) and contained the inactivated SRS or ISAv antigen encapsulated in a biological matrix licensed by Advance BioNutrition Corporation (ABN). Among the farms, 42% only gave one booster; 44% gave two boosters; and 14% gave more than two boosters. Each vaccine was administrated according to the specifications stipulated by the manufacturer.

### IgM purification

Twenty healthy and unvaccinated 30–50 g Atlantic salmons were obtained from a local aquaculture facility and housed in Virbac-Centrovet facilities at a density of 15 kg/m^3^ in tanks of 0.1 m^3^. To obtain the serum, fish were anesthetized in benzocaine 0.001% v/v solution and bled through the caudal vein. Blood samples were left overnight at 4°C and then centrifuged for 2 min at 8000 rpm (7168 RCF). The sera were dialyzed against sterile phosphate buffered saline solution (PBS – pH 7.4, 0.1M) and stored at −20°C until use. Finally, IgM was separated by size-exclusion chromatography (SEC) using sephacryl-S300 High-Resolution medium (GE Life Sciences – GE Healthcare, Sweden). The resin was hydrated in distilled water and packed into a 100 mL glass burette. The column was washed twice with 300 mL distilled water and equilibrated with PBS using a peristaltic pump with constant flow rate of 0.5 mL/min. The serum (15 mL) was dialyzed against sterile PBS before its loading, the elution fractions were collected every 1 min (0.5 mL).

### Dot blot analysis

Different elution fractions from SEC were placed onto nitrocellulose membrane and blocked overnight at 4°C with 2% skimmed milk-in PBS. The membrane was washed three times with PBS-Tween20 0.02%, incubated for 2 h at 37°C with mouse monoclonal anti-salmon/IgM antibody (Clone 3H7/E1, diluted 1:1000 – GrupoBios, Chile) and again washed three times with PBS-Tween20 0.02%. Finally, the membrane was incubated with an anti-mouse IgG conjugated with peroxidase for 1 h at 37°C (diluted 1:2000 – Sigma), washed three times and developed with the ImmPACT DAB Kit SK-4105 kit (Vector Labs).

### HPLC of IgM fraction

Positive IgM fractions were analyzed by HPLC using an equilibrated Atlantis Waters/250 mm × 10 mm (10 μm)/dC18 column (Atlantis Columns) in Prominence LC-20A-HPLC-04 equipment (Shimadzu, Japan). The sample was injected (20 μL) and eluted under isocratic conditions, acetonitrile/H_2_O 60:40. The column was operated at 2.0 mL/min flow rate, 27°C and 15 bar. The presence of proteins was observed at 280 nm. The purified IgM protein was quantified using a NanoQuant Infinite M200Pro spectrophotometer (Tecan Group Ltd.).

### IgM ELISA

To quantify the IgM concentration present in the serum of vaccinated fish, a standard curve using the purified IgM was developed. Briefly, serial twofold dilutions of purified IgM in carbonate buffer (NaHCO_3_ 0.2M, pH 9.6) were incubated in 96-well polystyrene plates (Thermo Scientific) at 37°C for 2 h. To detect specific anti-*P. salmonis* and anti-ISAv specific antibodies on vaccinated fish, the outer surface protein A (OspA) from *P. salmonis* and the hemagglutinin (HA) and neuraminidase (NA) from ISAv were used as recombinant antigens to coat the plate (4°C – overnight), respectively. Unbound antigens or IgM were removed with PBS-Tween20 0.05%. The plates were blocked with 2% skim milk-PBS for 2 h at room temperature (25°C). After blocking, microplates were washed three times and 1/50-fold dilution of each serum sample was added to the wells. Plates were then washed and incubated with a mouse monoclonal anti-salmon/IgM antibody for 2 h at 37°C (Clone 3H7/E1, diluted in PBS 1:1000 – GrupoBios, Chile) and later with a horseradish peroxidase-conjugated goat anti-mouse IgG antibody for 1 h at 37°C (diluted in PBS 1:2000). Finally, the plates were developed using 3,3,5,5′-Tetramethylbenzidine (TMB, 1 mg/mL in DMSO) at room temperature during 10 min and the reaction was stopped with 2M sulfuric acid. The absorbance was read at 450 nm using the Expert Plus ELISA reader (Asys).

### SRS mortalities in the field

The field data were obtained from a Chilean farm located in the X region of Chile. *S. salar* was grown in 26 cages of 50,000 fish each with an average weight of 1500 g. The vaccination program of the farm established two vaccinations (injectable and oral) against *P. salmonis*. SRS mortalities during the productive cycle were evaluated by PCR and symptomatology.

### Statistical analysis

The results of the experiments were expressed as the means ± SE. Comparisons between groups were made using One-way ANOVA – Dunnet post-test. Statistical significance was defined as a *p* value smaller than 0.05. Analyses were performed using GraphPad Prism software (USA).

## Results

### Quantitative ELISA for salmon IgM determination

**Figure 1:**
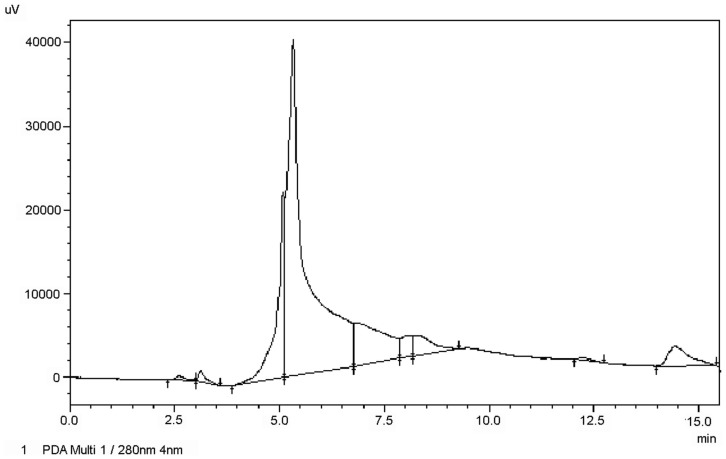
**Chromatography of purified IgM from *S. salar***. The IgM of Atlantic salmon previously separated by SEC was subjected to HPLC on dC18 column (Waters) and eluted under isocratic conditions. The chromatography was monitored at 280 nm.

To quantify the antibody levels present in the serum of fish, the IgM protein from *S. salar* was isolated and purified in order to generate a quantitative ELISA assay to allow the conversion of absorbance values into specific IgM concentrations. Serum samples from vaccinated Atlantic salmon were purified and prepared to analysis by SEC. The presence of the IgM protein in the various fractions was determined by a dot blot assays using a specific monoclonal anti-IgM antibody. Dot blot analysis indicated that the third eluted fraction contained most of the IgM protein (Data not shown). This fraction was further analyzed and compared by HPLC to determine its purity. The chromatograms in Figure 1 show that the purified IgM protein presents one homogeneous main signal at an elution time of 5 min. Finally, the IgM protein was quantified and used as a standard in the ELISA test.

### Detection of *P. salmonis* and ISAv specific IgM antibodies on vaccinated fish

**Figure 2:**
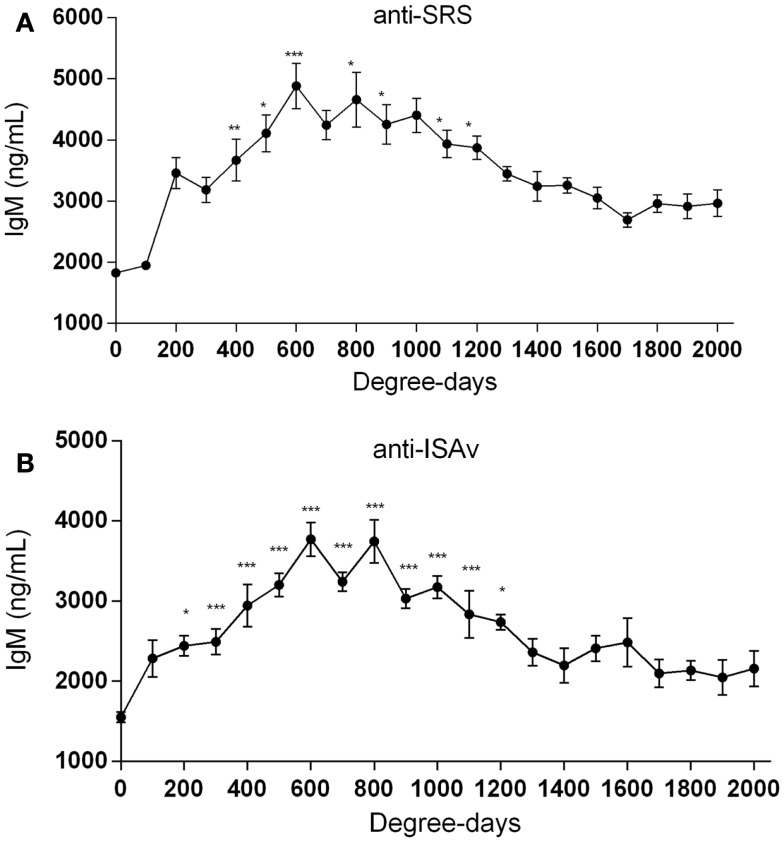
**Injectable vaccination increases IgM antibody titers**. Salmonids from different aquaculture industries were immunized i.p (0.1 mL/fish) with injectable mono or polyvalent vaccines against SRS **(A)** or ISAv **(B)** from either Centrovet or other pharmaceutical companies. The IgM concentration was followed up to 2000 degree-days. Serum samples were obtained at different degree-days to determine specific anti-*P. salmonis* and anti-ISAv antibodies through a quantitative ELISA assay. The samples were statistically analyzed by one-way ANOVA – Dunnet post-test. (SRS 120 fish/point, ISAv 65 fish/per point) *p < 0.05; **p < 0.01; ***p < 0.001.

**Figure 3:**
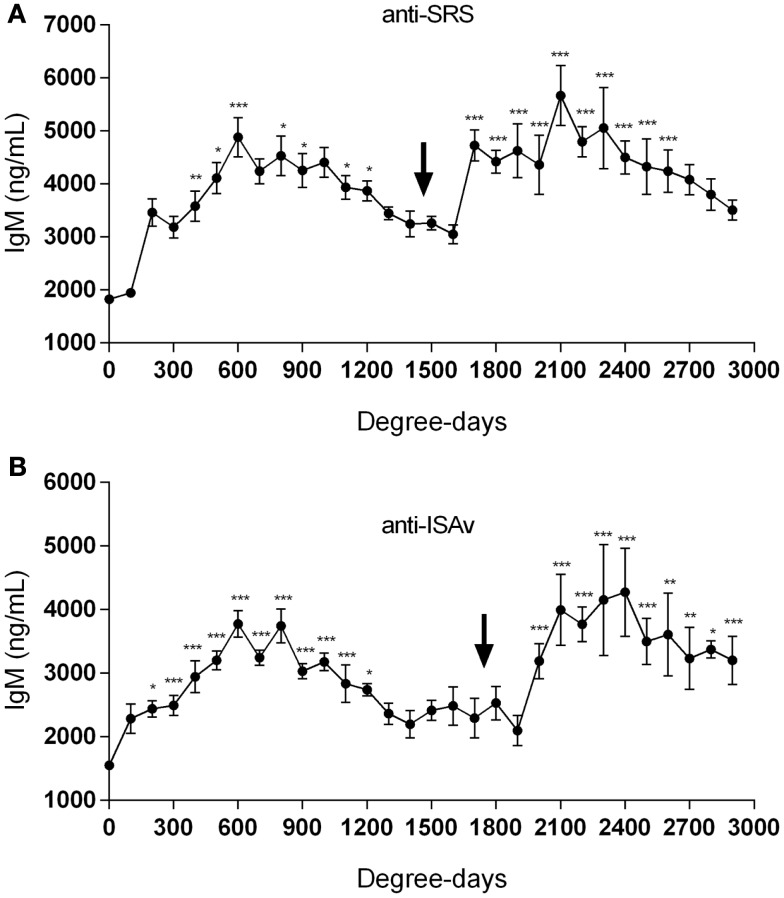
**Oral immunizations increase antibody production**. Fish were immunized by injectable vaccines as primo-vaccination and later as booster immunization by the oral route against SRS **(A)** and ISAv **(B)** infections. The IgM level was followed up to 3000 degree-days. The arrow indicates the time-point where the oral vaccine was administrated. Samples were statistically analyzed by one-way ANOVA – Dunnet post-test. (SRS 120 fish/point, ISAv 65 fish/per point) *p < 0.05; **p < 0.01; ***p < 0.001.

**Figure 4:**
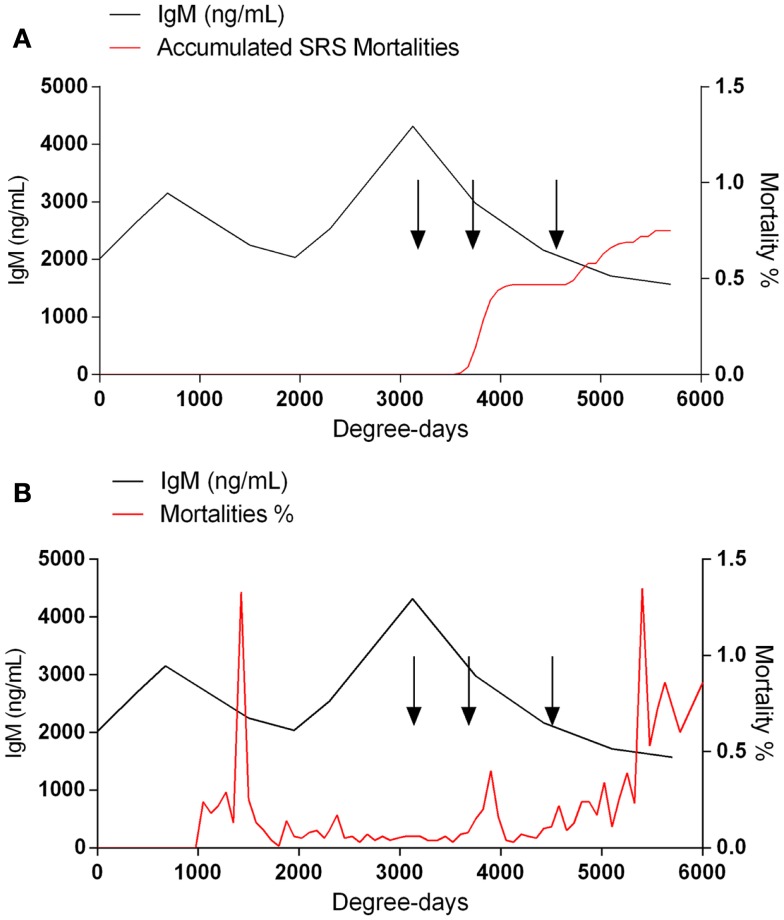
**A decreased IgM concentration is associated with an enhanced susceptibility to SRS infection**. The IgM concentration and the mortality rate were monitored on daily basis during the productive cycle. Fish with SRS **(A)** or overall mortalities **(B)** was analyzed. SRS mortalities were diagnosed by PCR and symptomatology. The arrows at 3200, 3700, and 4600 degree-days indicate florfenicol administration.

**Figure 5:**
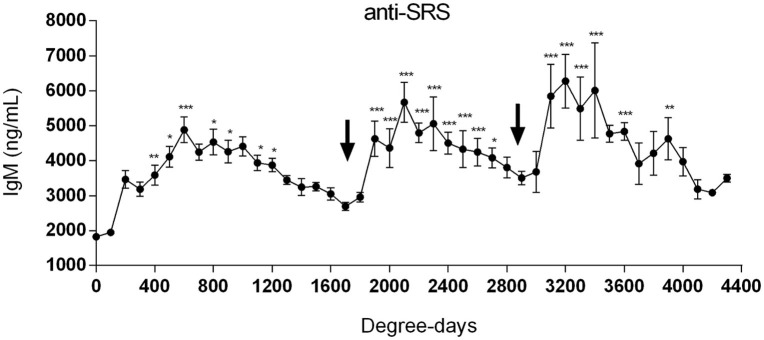
**Several oral immunizations are required to maintain a long-term protection against SRS**. Salmonids from different aquaculture industries were first immunized with an injectable mono or polyvalent vaccine against SRS. The arrows at 1700 and 2900 degree-days indicate the time-point where first and second oral vaccines were administrated, respectively. Serum samples were obtained at different degree-days to determine specific IgM anti-*P. salmonis*. Samples were statistically analyzed by one-way ANOVA – Dunnet post-test. (SRS 150 fish/point, ISAv 70 fish/per point) *p < 0.05; **p < 0.01; ***p < 0.001.

When the sera collected from fish at the different aquaculture farms (immunized with a primary injection of a commercial mono or polyvalent vaccine by i.p against SRS or ISAv) were analyzed to detect anti-*P. salmonis* and anti-ISAv antibodies, the results showed that specific IgM antibodies against both pathogens increased significantly after vaccination. The highest IgM concentration post vaccination was observed between 600 and 800 degree-days (Figures 2A,B). Interestingly, after 1300 degree-days, the IgM level against SRS and ISAv was not significantly different from unvaccinated fish. A second immunization had to be performed between 1300 and 1700 degree-days in order to maintain a high-protective IgM level. The results indicated that oral immunizations, given as a booster vaccination, rapidly increased concentration of specific IgM antibodies in vaccinated fish, maintaining the antibody response up to 2800–3200 degree-days (Figures 3A,B). In that context, we studied the mortality rate against SRS from one aquaculture center that performed two immunizations, injectable and oral booster. The analysis showed that both immunizations maintained a high level of antibodies against SRS up to 3600 degree-days. However, when the IgM concentration induced by the second vaccination decreased (1500–2500 ng/mL), SRS and overall mortalities began to increase (Figures 4A,B). In addition, an antimicrobial treatment was applied close to the window of susceptibility in order to maintain the protective state when the IgM concentration was decreasing. The results showed that the antibiotic treatment did not prevent the increase of SRS mortalities. In order to avoid the low concentration of antibodies and the susceptibility window to SRS after oral booster, some farms performed a third immunization (second oral). The vaccinated fish rapidly augmented the IgM concentration up to an average of 6000 ng/mL between 3200–3400 degree-days; however, the antibody titer and the duration of the immunity diminished at 4000 degree-days (Figure 5).

## Discussion

Vaccination is a cost-effective method for controlling infectious diseases in aquaculture such as SRS and ISAv, and has been demonstrated to significantly reduce disease outbreaks during production. The study of the immune response of vaccinated fish has been an area of particular interest in the last years. Indeed, there is a growing demand to establish the protective status of fish and determine how this correlates with immunological signatures after vaccination to provide evidence of the efficacy and the protective state conferred by vaccines (11). This is important not only at scientific level but also at productive level especially to reduce the cost/benefit ratio of massive vaccination programs. The efficacy of vaccination has been often attributed with specific antibody levels present on immunized fish.

Although, correlate of protection against SRS and ISA is a topic of discussion by the industry in Chile, recent studies have shown that there is close association between specific IgM antibodies and vaccine protection (12). Recently, it was demonstrated that relative percent survival (RPS) values can be associated with antibody titers induced by vaccination (5). In the present study, we have shown that to reduce SRS and ISA mortalities, several immunizations are necessary in order to maintain a high concentration of specific IgM antibodies. We determined that concentrations below 2000 ng/mL of specific anti-SRS IgM antibodies generate a susceptibility window that increases the probability of SRS outbreaks. On the other hand, concentrations above 2500 ng/mL were able to confer protection from infection, indicating the close association between protective antibodies and disease outbreak. Interestingly, antibiotic treatment did not prevent SRS mortalities, indicating that vaccination is crucial to avoid losses, especially against antibiotic resistant *P. salmonis* strains. Although the percentage of SRS deaths was low, fish entered into susceptibility window where other infections such as bacterial kidney disease (BKD), IPN, caligus, and fungi may act (Figure 4). The high-antibody level after vaccination together with the reduction of disease outbreaks have been observed in several farms in Chile during three years of the following up service.

The IgM analyses of vaccinated fish were consistent with the notion that, in the field, injectable vaccines do not protect over the entire productive cycle, which in our study was around 800 degree-days. Similarly, booster immunizations by oral administration have shown excellent results under experimental conditions (1, 13). The success of oral vaccination is closely related with the amount of antigen that is internalized and processed by fish (14). Thus, oral vaccines have to protect the antigen from protease degradation and gastric pH in order to activate the immune system in the gut and other mucosal tissues. Our results indicated that oral immunizations promoted a similar increment of antibody titers in comparison with injectable vaccine. In contrast to injectable vaccines, oral vaccination has the advantage that it can be used in large-scale procedures and during the complete sea-water period, being an effective way to maintain the protective state along the entire productive cycle (15).

The short-term protection induced by injectable and oral vaccines in the field might be explained by the unique immune system of salmonids. In teleost fish, the B cell response presents different dynamics in terms of antibody response, affinity maturation and immunological memory. The ability to generate highly specific antibodies associated with the process of affinity maturation occurs relatively late in the antibody response (16–19). The immunological memory is one of the hallmarks of vaccination and it is crucial to induce long-term protection. In mammals, the immunological memory depends mostly on the presence of memory B cells and the persistence of long-lived plasma cells (LLPC) (20). In salmonids, it has been established that memory B cells response occurs but in a much lesser degree in comparison to mammals. Antigenic re-stimulation of these cells promotes only arithmetic but not logarithmic increase of the antibody titers upon second challenge with thymus-dependent antigens (21). In the same line, our results showed that upon booster immunizations the antibody titers tend to increase arithmetically in comparison with the first immunization. Recently, it was demonstrated that the existence of LLPC in the anterior part of the kidney in response to thymus-dependent and -independent antigens (15). These cells provide a persistence humoral immune response against pathogens due to the sustained liberation of high-affinity antibodies (22, 23). The variability and limited duration of antibody response after vaccination observed in this study may be due to the lack of the appropriate physiological and environmental conditions such as chemokines, cytokines, and cell-to-cell contact, required to maintain these cells within the anterior kidney (24, 25). Additional studies have to be done in order to understand more the immunological memory in teleost fish. In addition, the short-term protection promoted by vaccination indicated that extrapolations of experimental conditions on fish immune response to commercial productive conditions are difficult to accomplish. The maintenance of a comprehensive vaccination program will be crucial to prevent or nullify the risk or susceptibility to infectious diseases. The knowledge that successive immunizations are essential to maintain a protective state until harvest on farmed salmonids will help producers to enhance existing sanitary conditions and also to have a higher yield and quality in their productions. Other analytics development such as the identification and detection of different lymphocytes populations, cytokine profiles, and polarization of the immune response and IgT determination will improve decision-making and in consequence overall productivity of the farms.

## Author Contributions

Conceived and designed the experiments: IT and JT. Fish sampling: MA and EN. Performed the experiments: IT, CT, VV, PS, and CC. Analyzed the data: SA and IT. Wrote the manuscript: SA and JT.

## Conflict of Interest Statement

The authors report no conflict of interest. The authors alone are responsible for the content and writing of the paper.
